# Effects of hsa-mir-145-5p on the Regulation of msln Expression in Colorectal Adenocarcinoma

**DOI:** 10.1155/2022/5587084

**Published:** 2022-03-15

**Authors:** Junhua Li, Bulin Baila, Tian Xiang Xu, Jiang Song, Su Rina, Ji Wu, Tegexibaiyin Wang

**Affiliations:** ^1^PLA Research Institute of General Surgery, Jinling Hospital, The First School of Clinical Medicine, Southern Medical University, Nanjing, 210002 Jiangsu, China; ^2^Pharmacy Laboratory, Inner Mongolia International Mongolian Hospital, No. 83 Daxuedong Road, Hohhot 010065, China; ^3^Abdominal Tumor Surgery, Inner Mongolia People's Hospital, No. 20 Zhaowuda Road, Hohhot 010017, China

## Abstract

Colorectal cancer (CRC) is one of the most common gastrointestinal cancers in the world, and its incidence is increasing all over the world including China. In recent years, research data show that some miRNAs are differentially expressed in cancer tissues, and their expression is closely contributed with the prognosis of CRC. Microarray technology was used, and 179 miRNAs were screened out with significantly altered expression in CRC tissues compared with adjacent tissues. The expression of mir-145-5p in tumor tissues was 3.48 times lower than that in normal tissues. Using bioinformatics technology and network resource prediction, we found that mir-145-5p had a potential target gene relationship with *msln* gene. Then, qRT-PCR was used to validate the expression level of mir-145-5p and *msln* mRNA in CRC and paracancerous tissues. The results showed that *msln* mRNA was higher than in normal tissues, while mir-145-5p was lower, with statistically significant difference (*P* < 0.01, *n* = 3). Furthermore, the expression of msln protein in CRC and normal colorectal tissues was detected by protein mass spectrometry (MRM) (*n* = 3) and immunohistochemistry in a total case of 30 colorectal cancer tissues and normal tissues. Result showed that the positive expression of msln in CRC was higher than that in normal colorectal tissues, 1.38*e*-6 and 1.89*e*-6, respectively (*P* < 0.01, *n* = 3). Furthermore, in 48 h RTCA real-time monitoring experiment, mir-145-5p showed inhibitory effect on the proliferation of colo320 cells stimulated by msln. This study demonstrated that *msln* is a target gene of mir-145-5p in CRC. Besides, mir-145-5p negatively regulates the proliferation of CRC colo320 cells through downregulating *msln* gene expression in CRC colo320 cells.

## 1. Introduction

Colorectal cancer (CRC) is one of the most common gastrointestinal malignant tumors in the world, and incidence is increasing every year in many countries including China [1]. Although the diagnosis and treatment level of colorectal cancer has been continuously improved, its five-year survival rate has not been significantly improved. Therefore, it is a hot research topic to further clarify the exact molecular mechanism of colorectal cancer development [2, 3]. The exact mechanism for the development of colorectal cancer has not yet been fully elucidated. A large number of studies have shown that it may be related to oncogenes, maladjustment of tumor suppressor genes, changes in cell signaling pathways, gene polymorphisms, and microsatellite instability [4, 5].

Colorectal cancer is mainly adenocarcinoma, and the rest is mucous adenocarcinoma and undifferentiated carcinoma. The general form can be polyp and ulcer. Colorectal cancer can develop along the wall of the intestine, spread up and down the longitudinal diameter of the intestine or infiltrate deep into the intestinal wall. In addition to lymphatic tube, blood flow transfer and local invasion, it can be planted in the abdominal cavity or spread along sutures and incisions. It is more common in middle-aged men, from adenoma polyp cancer, the onset of younger age [5, 6]. The main causes of colorectal cancer are diet, smoking, drinking, lack of physical activity, family inheritance, colon polyps, and exposure to radiation. The survival rate of colorectal cancer patients has not been satisfactory despite the use of surgery, chemotherapy and radiotherapy. Therefore, the medical community hopes to find a new treatment method that can more effectively improve the effective cure rate of colorectal cancer [7].

Liebig et al. found the high expression of mesothelium in the blood and tissues of patients with colorectal cancer. However, its function was not discovered. Therefore, our group predicted that mesothelioma is likely to play an important role in the development of colorectal cancer [8, 9].

Mesothelin is a protein that produces the mesothelin (msln) gene. Many scientists believed that mesothelioma was a specific protein that was expressed only in mesothelioma. However, recent studies have shown that more than 70% of cancer patients express mesothelin in their tissues, including colorectal cancer, lung cancer, stomach cancer, mesothelioma, ovarian cancer, and pancreatic cancer [10, 11]. The mesothelium gene is inhibited in normal tissues and activated when cancer occurs. So far, the good versatility of mesothelium has not been discovered. Some scholars believe that mesothelin may participate in cell adhesion and stimulate the growth of cancer cells [12–14].

On the other hand, although thousands of miRNAs have been found to be involved in tumor, the molecular biological functions and mechanisms of most of them in tumors are still unknown, and reports in the field of colorectal cancer are more limited [15, 16]. Currently, microRNA-145 (mir-145) expression was found to be reduced in mesothelioma and closely related with the occurrence of mesothelioma [3–5]. As a newly discovered microRNA, mir-145 was found to have significantly low expression in various tumors [17–21]. msln is one of the genes that are not expressed in normal tissues and are highly expressed after cancer occurs. The literature shows that it plays an important role in the process of tumor development, invasion, and metastasis. The colorectal cancer has the malignant biological behavior of local invasion and cervical lymph node metastasis, which is the focus of this topic.

Since miRNAs are widely involved in the occurrence and development of tumors by inhibiting target genes, we speculate that those highly expressed protein msln in colorectal cancer may be related to some miRNAs with reduced expression.

To verify this hypothesis and further explore the biological significance of decreased expression of mir-145-5p in colorectal cancer, we use miRNA expression profiling chips to determine the expression profiles of miRNA in colorectal cancer tissues and normal intestinal tissues.

Then, the target regulation relationship of mir-145-5p to the msln 3′-UTR area was discussed [1, 21–24]. We also screened the expression reduction of miRNAs in colorectal cancer tissue using high-throughput detection [25]. It was observed that msln and mir-145-5p have strong target binding possibilities through bioinformatics prediction.

Finally, the relevant analysis data was further confirmed by qRT-PCR. Also, the effect of mir-145-5p was determined in vitro by transferring mir-145-5p into colo320 cells, using a multifunctional real-time unmarked cell analyzer (xCELligence RTCA DP, RTCA) analysis system with real-time monitoring of colorectal cancer colo320 cell activity. The effects of mir-145-5p on the proliferation and metastasis of colorectal cancer cells through the regulation of the potential target gene *msln* were elucidated [26–28].

## 2. Methods

### 2.1. Ethics Statement

All participants in this study provided written consent. All experiments and analyses in this study were approved by the ethics committee of the Inner Mongolia People's Hospital (reference number 3217–08).

### 2.2. Patient and Public Involvement

From March 2016 to December 2017, 30 cases of primary colorectal cancer without radiotherapy, chemotherapy, or other tumor-specific treatment were collected in the People's Hospital of Inner Mongolia Autonomous Region. All the patients had abdominal pain and bloody stool. There were 18 females and 12 males, all aged between 45 and 70 years old. The samples of each case included a part of the lesion and a part of the adjacent normal colorectal tissue more than 5 cm away from the lesion. The collected patients with primary colon cancer had no primary tumor in other parts; no history of cardiovascular and cerebrovascular diseases such as diabetes and hypertension; no history of hepatitis B, syphilis, tuberculosis, and other infectious diseases; and no family history of genetic diseases. The samples were collected by aseptic operation within 5 minutes in the operating room and stored in liquid nitrogen rapidly.

### 2.3. Immunohistochemical Staining Test

Human colorectal cancer tissues were fixed with 4% paraformaldehyde solution, embedded in paraffin, and stained with conventional histology. The slices were dewaxed in xylene, dehydrated in ethanol, and heated with citrate for 15 min; then, the thin-walled slices were taken out and treated with 0.3% hydrogen peroxide for 15 min to block the endogenous peroxidase activity. The sections were further sealed with 2% BSA and then incubated with rat monoclonal antibody against msln (sc-33672, Santa Cruz) at 4°C for more than 16 hours. After washing, the second antibody (g1210-2-a, Servicebio) was incubated at room temperature for 60 min. The expression of msln protein was observed by DAB staining. The slides were redyed with hematology before dehydration and mounting. All sections were observed and photographed under fluorescence microscope (Nikon, Japan).

### 2.4. Total RNA Extraction

The total RNA was extracted using TRIzol from tissue specimens of colorectal cancer patients and separated from colorectal cancer cells. The amount was quantified and stored at -80°C.

### 2.5. Screening of MicroRNAs with Different Expressions

Total RNA extracted from 3 cases of colorectal cancer and its corresponding adjacent normal tissues was selected, and miRNA expression in 6 different tissues was detected by Illumina microRNA chip technology. Then, the hybridized chip was scanned and signal was extracted by Illumina scanner.

### 2.6. Original Data Analysis Using GenePix Pro V6.0 Software

After the green signal intensity of each probe on the chip was debackgrounded, the average of four replicate probes was obtained. The raw data were normalized by median normalization, and the standardized data was obtained. In a batch of experiments, the noncontrol probe with a correction value (foreground value-background value) of ≥50 on each chip was normalized, and the median of this part was used as a normalization factor for the point of the whole chip. Then, standardized processing was carried out for each: miRNA correction value/median value = standard value. After standardization, differentially expressed miRNAs were calculated by statistical *t*-test method. Finally, unsupervised clustering and correlation analysis were performed on miRNA chip data.

### 2.7. Prediction of Target Genes

In order to predict the target genes of miRNAs by theoretical methods, the target bioinformatics software TargetScan, PicTar-Vert, and MiRanda were used.

### 2.8. Statistical Processing

The gene chip data was processed by Agilent Feature Extraction software. After the data was homogenized, the differential expression of a certain miRNA in tumor and normal tissues was expressed. The difference was 3 times and above, and the statistics were collected from 3 pairs of samples and clusters. miRNAs were upregulated or downregulated consistently between the two groups (HJ-1, HJ-2, HJ-3, HJC-1, HJC-2, HJC-3). The miRNA data with valid signals detected in 3 tissues were clustered by homogenization, cluster analysis was performed using Cluster 3.0, and the clustering tree was drawn by TreeView software. The relative gene expression intensity was expressed as mean ± standard deviation. The difference between groups was compared by *t*-test, *a* = 0.05. Correlation analysis was performed using Pearson correlation and calculated by Stata10 statistical software.

### 2.9. Cell Culture and MicroRNA Transfection

The cells were seeded in a 6-well plate at a concentration of 1 × 10^6^ cells/well and allowed to grow to the logarithmic growth phase. Cells were transfected when grown to 60%-70% of the culture dish. The experiment was divided into 4 groups: control group, has-mir-145-5p group, msln group, and has-mir-145-5p plus msln group. Prepare four sterile 1.5 ml EP tubes, add 5 *μ*l lipofectamin 2000, 10 nmol/L mir-145-5p mimics, pcDNA3.1-msln, and mir-145-5p+pcDNA3.1-msln into each tube. Then, mix well and let stand at room temperature for 20 min. The culture medium in the 6-well plate was removed by aspiration. 1 ml of serum-free RPMI1640 medium was added to each well, and then, the transfection mixture was added to each well to cells and mixed carefully. The cells in the transfection mixture were cultured in a CO_2_ incubator at 37°C for 4 to 6 hours. The transfection mixture was removed after transfection. 1.5 ml of RPMI1640 medium containing 10% FBS was added to each well and continued to be incubated. After 12 hours, the cells were collected for the next experiment.

### 2.10. qRT-PCR Validation

The expression levels of hsa-mir-145-5p and *msln* mRNA in CRC were detected by qRT-PCR, and the expression levels of nine miRNAs including hsa-mir-574-5p, hsa-mir-141-3p, hsa-mir-200c-3p, hsa-mir-4489, hsa-mir-125b-5p, hsa-mir-143-3p, hsa-mir-1-3p, and hsa-mir-1281 were preliminary detected.

The primer sequence was as follows: hsa-mir-145-5p forward: 5′-GTCCAGTTTTCCCAGGAATC-3′, reverse: 5′-GATTCCTGGGAAAACTGGAC-3′; internal reference *β*-actin: forward: 5′-CTG GGA CGA CAT GGA GAA AA-3′, reverse: 5′-AAG GAA GGC TGG AAG AGT GC-3′; and human mesothelin: forward: 5′-ACCGACGAGGAACTGAATGCTCTT-3′, reverse: 5′-ACGATGGACTCATCCAACACTGCT-3′. The PCR reaction conditions were as follows: predenaturation at 95°C for 5 min, 95°C for 30 s, 56°C for 30 s, 70°C for 30 s, 35 cycles, and 72°C for 10 min. Three replicate wells were set in each group and repeated three times. The gene expression level was calculated according to the 2^−*ΔΔ*Ct^ method. The results were analyzed using Quantity One 4.6.2 software.

### 2.11. Western Blot

The msln protein expression in colo320 cells was measured by Western blot. Briefly, 100 *μ*l lysate [50 mM Tris-HCl (pH 7.4), 150 mM NaCl, 1% NP-40, 0.1% sodium dodecyl sulfate (SDS)] was added to the cultured cells, shaken violently for 2 min, incubated on ice for 20 min, and then centrifuged at 13,000 rpm (4°C) for 20 min. Taking the supernatant and the protein content was determined according to the instructions of the BCA protein quantitative kit. Then, aliquots of 50 *μ*g homogenate protein were run on 10% (*w*/*v*) SDS-polyacrylamide gel electrophoresis (PAGE) gels. Proteins were subsequently transferred to nitrocellulose membranes, which were blocked by incubation with 3% (*w*/*v*) fat dry milk in PBS for 1 h at room temperature, with shaking. Thereafter, the membranes were incubated overnight at 4°C with rabbit polyclonal anti-msln (sc-33672, Santa Cruz) or rabbit polyclonal antiactin (Santa Cruz, Biotechnology, Santa Cruz, CA, USA). The primary antibody was used at dilutions from 1 : 500 to 1 : 1,000. The blots were washed four times with Tris-buffered saline Tween-20 (TBST) and subsequently incubated for 2 h at room temperature in Tris-buffered saline with horseradish peroxidase-conjugated antirabbit IgG antibody (1 : 5,000; sc-33672, Santa Cruz). Gel Imaging Systems ver. 4.00 software was used to scan the image for gray analysis. Results were calculated as the relative ratio of the specific band compared with actin.

### 2.12. Multivariate Analysis of MRM Measurements

All protein quantification was tested by triplet of 5600 + mass spectrometer of AB company. The collision energy (CE) voltage was optimized, and the specific peptide segment of mesothelin protein was selected by Skyline software and optimized by increasing the voltage from - 5 V to +5 V around the predicted CEs. Three peptides of GAPDH protein were selected as internal reference for correction, and three specific peptides of mesothelin protein were selected for quantification. Therefore, the MRM capture parameters and retention time of the three peptides are the same. The only difference is the actual *M*/*Z* value of the precursor and product ion.

### 2.13. Chromatographic Conditions

Columns used were as follows: Eksigent C18 trap column (10 × 0.3 mm, 5 *μ*m) and Eksigent C18 analytical column (150 × 0.3 mm, 3 *μ*m). Mobile phase: 0.1% formic acid aqueous solution (A) : 0.1% formic acid acetonitrile solution (B) with gradient elution (0-1 min, 5%-6% B; 1-65 min, 6%-30% B; 65-70 min, 30%-50% B; 70-72 min, 50%-80% B; 72-80 min, 80% B; 80-80.5 min, 80%-5% B; 80.5-90 min, 5% B). Loading flow rate was 10 *μ*l/min and gradient flow rate was 5 *μ*l/min.

### 2.14. Mass Spectrometry Conditions

Mass spectrometer used was triplet of 5600+. IDA acquisition data: (1) First level parameters: *M*/*Z* region: 350-1500; accumulation time: 0.25 s; charge: +2∼+5. Source gas parameters: gas 1: 10; gas 2: 20; curtain gas: 30; TEM: 300; DP: 100; CE: 10. (2) Second level parameters: 50 data-dependent MS/MS scales per full scan; *M*/*Z* region: 100-1500; acquisition time, 0.03 s. Rolling CE method was used.

### 2.15. Protein Identification

Protein analysis was carried out by ProteinPilot 5.0 software. The database retrieval parameters are as follows: Proteins were identified according to Cys residues and alkylated. Proteins were then digested by trypsin. Instrument used was TripleTOF™ 5600. Swiss Prot-human (20367 entries) database was used to identify the proteins.

### 2.16. Determination of Cell Proliferation Activity by RTCA Method

The colo320 cells were cultured in a medium culture bottle, the has-mir-145-5p mimic and the PC DNA 3.1-msln plasmid were converted, 24 H cells were collected, and 1 × 10^4^/ml colo320 cells were inoculated on the Kongzhong 16 plates of the RTCA system. After the serum-free synchronous treatment of each hole, the growth of 0 to 48 H colo320 cells was dynamically monitored by the variation of microelectrical resistance in each hole of the RTCA system. The effects of has-mir-145-5p and msln on the proliferation of colo320 cells in colorectal cancer were studied by the kinetic change response curve (CI curve) reflecting cell proliferation.

## 3. Results

### 3.1. Screen of Differentially Expressed Genes in Colorectal Cancer Tissue

**Figure 1:**
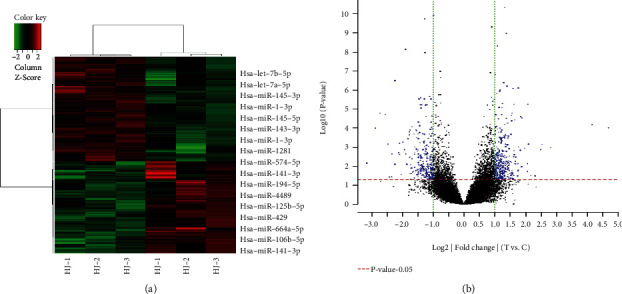
Gene coexpression network analysis. (a) Heatmaps of gene expression. Red is up, green is down. The darker red indicates that the expression is higher, while the darker green indicates that the expression is lower. It can be concluded that the samples can be divided into general clusters: false injury control group and injury experimental group. (b) Blue dots represent genes that have statistically significant differences in expression. The blue dot represents an increase in gene expression, while the blue dot on the left represents a decrease in gene expression. Black indicates that there is no statistically significant difference in gene expression. The larger the longitudinal coordinate value, the corresponding point is, the greater the difference in gene expression corresponding to this point. Similarly, the greater the absolute value of the transverse coordinate corresponding to the point is, the greater the difference in gene expression corresponding to the point.

Differentially expressed genes were screened according to the *P* values and fold changes firstly. Then, the similarity of overall gene expression between experimental samples was further analyzed using unsupervised hierarchical clustering. The data from the clustering analysis please refers to the file “Array QA/C lustering_analysis” and the cluster diagram “cluster_data.gif” which contains gene names. 179 differentially expressed genes were examined through cluster analysis of the quality of tumor tissue HJ (HJ1, HJ2, HJ3) and normal tissue HJC (HJC1, HJC2, HJC3) arrays in colorectal cancer (Figures 1(a) and 1(b)).

### 3.2. Identification of Mesothelin in Colorectal Cancer Tissues

**Figure 2:**
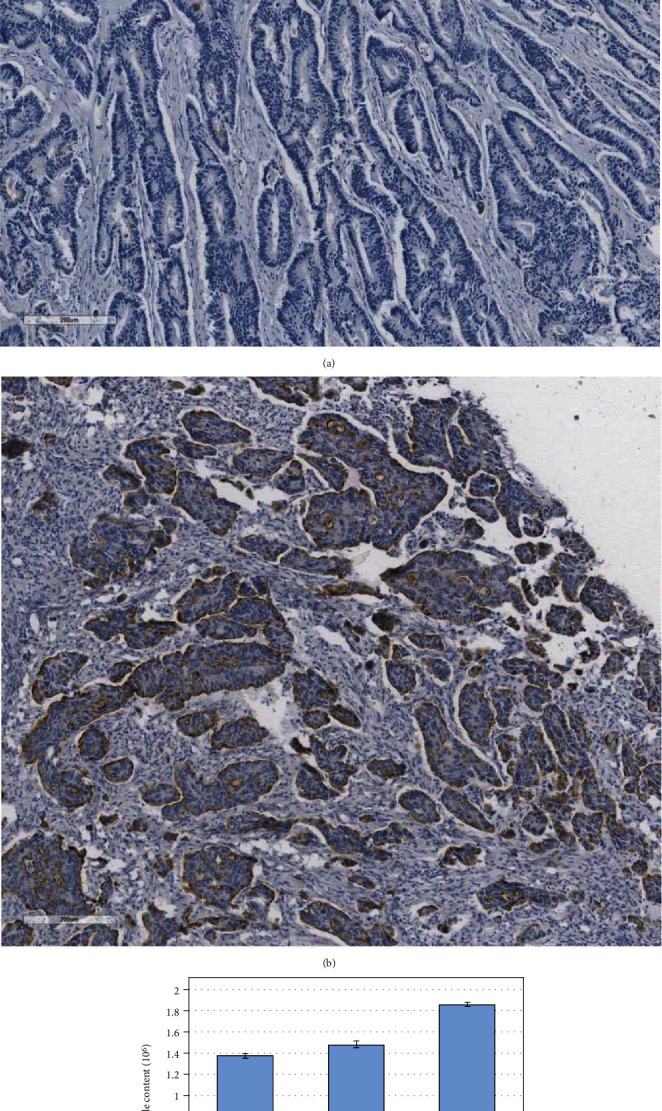
Identification of MSLN expression in colorectal cancer tissue by immunohistochemistry. MSLN was expressed 3% in pericancerous tissue (a) and 83% in colorectal cancer tissue (b). (c) Higher expression of MSLN was detected in colorectal cancer tissue than that in normal and pericancerous tissues by MRM method (*P* < 0.01).

Immunohistochemistry was used to detect the expression of msln protein in rectal adenocarcinoma specimens. Result showed that msln is mainly expressed on the cell membrane of colorectal adenocarcinoma (data not shown). In total case of 30 normal group (adjacent normal tissue of colorectal adenocarcinoma), msln was expressed only in 1 case. The positive rate was 3% (Figure 2(a)). However, the msln expression was observed in the colorectal adenocarcinoma tissue of 25 cases, accounting for 83% (Figure 2(b)), indicating that the msln expression in the colorectal adenocarcinoma tissue was significantly higher than that in normal tissues (*P* < 0.05). It is consistent with the report in literature.

In order to detect the msln protein, we used MRM measurement and selected three specific peptides of msln protein according to the uniqueness of the target protein and three peptides of GAPDH protein for internal reference. The selected peptide sequence covers GAPDH and msln. The best collision energy of each peptide was determined by experiments. The measurement of each peptide has the highest specificity and sensitivity for colorectal cancer. Sequence coverage (% COV) 100%; confidence >95% (% cov 95): 44.6%. The results showed that the expression of msln protein in rectal cancer was higher than that in normal and adjacent tissues (*P* < 0.01) as shown in Figure 2(c). It is consistent with the previous report that msln peptides was highly expressed in cancer tissues than that in normal tissues (Table 1).

### 3.3. Prediction of Target Gene

**Figure 3:**
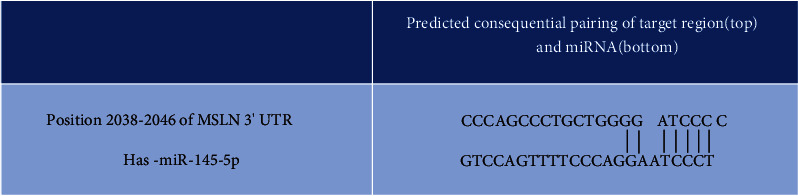
Prediction of the target of has-miR-145-5p through the binding site. Putative binding sites of has-miR-145-5p in the MSLN 3′UTR predicted by TargetScan.

In order to ensure the accuracy of prediction, MiRanda, miRDB, and TargetScan were used in this study. After screening based on this method, 179 genes were obtained as the target genes. It was further screened through PubMed database, and mir-145-5p was consequently found to be the potential target gene of msln which might be related to tumor, especially malignant tumor, including colorectal cancer occurrence, development, and malignant degree of correlation, according to the our result and the report. Meanwhile, the msln 3′UTR carries the binding site of mir-145-5P (Figure 3). It further indicates that mir-145-5p is the potential target gene of msln. Also, the expression of hsa-mir-200b-3p, hsa-mir-145-5p, and hsa-mir-143-3p showed significant difference in colorectal cancer and normal tissues (Table 2). This result indicated that msln is a target gene of mir-145-5P again.

### 3.4. Expression of mir-145-5p and msln mRNA in Colorectal Cancer Tissues

**Figure 4:**
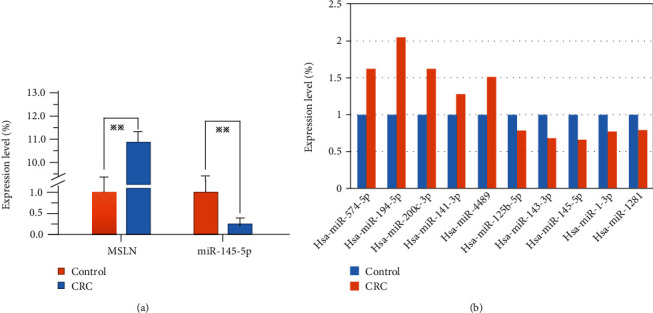
The relative expression levels of miR-145-5p and MSLN in 10 cases of colorectal cancer (CRC) and 10 cases of adjacent normal margin (control). (a) The relative expression levels of miR-145-5p and MSLN in 10 CRC tissues and 10 control tissues, respectively (*P* < 0.01). (b) Relative expression levels of CRC-related miRNAs in cancer tissues.

To investigate whether or not *msln* is a target gene of mir-145-5p, the expression of mir-145-5p and *msln* was firstly determined. In this study, qRT-PCR was used to detect the transcription levels of mir-145-5p and potential target gene *msln* in fresh tissues of 6 colorectal adenocarcinoma patients. The results showed that the average expression level of mir-145-5p in 3 cases of colorectal adenocarcinoma was 0.42 ± 0.78 times (*P* = 0.043) which was significantly downregulated compared to that in corresponding paracancerous normal tissues. Oppositely, the average expression level of *msln* in 3 cases of colorectal cancer was 12.50 ± 14.03 times higher than that in corresponding paracancer normal mucosal tissue (*P* = 0.029), which was significantly upregulated, as shown in Figure 4(a). Together with the previous result, it indicates that *msln* may be a direct target of mir-145-5P. Also, 9 other differentially expressed microRNAs, including hsa-mir-574-5p, hsa-mir-141-3p, hsa-mir-200c-3p, hsa-mir-194-5p, hsa-mir-4489, hsa-mir-125b-5p, hsa-mir-143-3p, hsa-mir-1-3p, and hsa-mir-1281 in CRC, were validated by qRT-PCR (Figure 4(b)).

### 3.5. The Effect of mir-145-5p on msln Protein Expression

**Figure 5:**
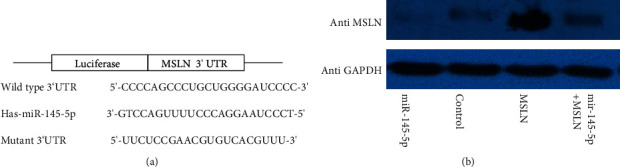
The addition of mir-145-5p inhibits MSLN protein expression in vitro. (a) Sketch of the construction of cDNA3.1-MSLN and miR-145-5p mimics. (b) Western blot results showed that in colo320 cells, the expression of MSLN protein was significantly increased in MSLN group, but it was inhibited in mir-145-5p and mir-145-5p+msln groups.

The effect of mir-145-5p on msln protein was determined in colo320 cells after being transfected with the plasmid for expressing msln and mir-145-5p mimics (Figure 5(a)) by Western blot. As shown in Figure 5(b), in colo320 cells, in the group transfected with msln-expressing plasmid, the msln protein was significantly increased compared to that in the control. But in the group transfected with mir-145-5p mimics + msln plasmid, the expression of msln was significantly inhibited. Also, in the group transfected with mir-145-5p mimics, the expression of msln was inhibited compared to that in the control group. It indicates that mir-145-5p inhibits the expression of msln protein in colo320 cells.

### 3.6. The Effect of mir-145-5p on the Activity of msln colo320 Cell Proliferation

**Figure 6:**
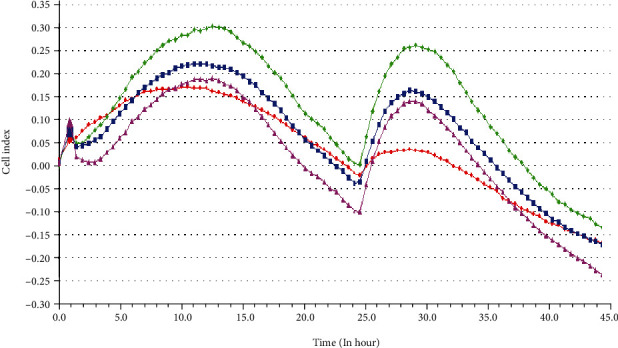
Real-time monitoring results of RTCA analysis system. The time from inoculation of E-Plate 16-hole plate to the start of synchronization; without serum culture, RTCA monitored the effects of miR-145-5p and MSLN on the proliferation of COLO320 cells in colorectal cancer. The control group, MSLN group, and mir-145-5p group were compared with the mir-145-5p+MSLN group, and there was statistical significance between the two groups (*P* < 0.01).

In a total of 48 h real-time monitoring, mir-145-5p mimics and msln-expressing plasmid alone transfected at the same time can inhibit the growth of colo320 cells. Both 10 nmol/l mir-145-5p and msln showed obvious effect on inducing cell proliferation. However, with the prolongation of the action time of mir-145-5p and msln, the CI curve tended to be consistent after incubation for 48 h. Therefore, 10 nmol/l and 12 h were the optimal concentration and treatment time of mir-145-5p and msln on the proliferation of colo320 cells. In addition, when colo320 cells were added into the system for less than 5 h, the cells showed stress response, which was manifested in the increase of cell growth curve reactivity. But with the extension of incubation time, the cell curve tended to decline, more accurately represented the real growth state of cells. After 48 h of transfection, the cell proliferation activity was relatively reduced. Compared with the control and msln groups, the relative cell proliferation activity of mir-145-5p plus msln decreased (76.47 ± 2.74%); compared with the mir-145-5p group, the cell proliferation activity of mir-145-5p plus msln decreased (36.50 ± 1.59%) as shown in Figure 6, suggesting that mir-145-5p can prevent the activity of msln in colo320 cell proliferation.

Together, these results indicated that *msln* is the target gene of mir-145-5p, which can inhibit the cancer cells proliferation through preventing the expression of msln at both gene and protein levels.

## 4. Discussion

The basic characteristics of tumor biology are invasion and metastasis which are the main reason causing tumor recurrence and death for most patients with tumor [1, 23]. Tumor cell metastasis is usually accompanied by regulation disorder of oncogene and tumor suppressor.

In recent years, miRNA studies have shown that the differential expression of some miRNAs in tumor tissues plays an important role in the prognosis of colorectal cancer, through regulating apoptosis, proliferation, neural development, and stem cell differentiation. It is reported that the expression level of mir-145-5p is low in many kinds of cancers, such as ovarian cancer. However, the up regulation of mir-145-5p can inhibit the invasion, metastasis and proliferation of cancer cells. Ling Wang et al. investigated that MUC1 is the direct target gene of mir-145-5p in ovarian cancer. mir-145-5p can inhibit the expression of MUC1 protein through complementarily binding with MUC1, so that can enhance the expression of E-cad, which is a marker protein of ovarian cancer. The high expression of E-cad can prevent exfoliation and metastasis of cancer cells. It indicates that mir-145-5p can block the invasion and metastasis of ovarian cancer cells by mediating the MUC1/E-cad signal pathway. Conversely, the effect of mir-145-5p was unregulated or inhibited by MUC1, suggesting that MUC1 is the target gene of mir-145-5p in ovarian cancer.

The msln protein is the primary product encoded by the mesothelin (msln) gene. The precursor protein is 71 kDa, and the cleavage c-erc/mesothelin is 40 kDa, which binds to the cell membrane, while n-erc/mesothelin (31 kDa) is secreted into the blood. Currently, the expression frequency of the msln protein in CRC samples was 40% (16 of 40 cases), 70% in ovarian cancer, 50% in lung cancer, and 46% in esophageal cancer, and in almost all mesothelioma and pancreatic cancer [1]. The msln protein presents in normal mesothelial cells and is overexpressed in several human tumors, including mesothelioma, ovarian cancer, lung cancer, and pancreatic cancer [1].

In this study, we focused on the relationship between mir-145-5p and msln protein in colorectal cancer. Firstly, the expression of mir-145-5p was detected in the middle bottom of colorectal cancer, and the high expression of msln in colorectal cancer was confirmed. Then, it was found that mir-145-5p had a negative regulation effect on the invasion and migration of colo320 cells. The upregulation of mir-145-5p can inhibit the proliferation, invasion, and migration of colo320 cells, as well as the expression of msln protein in colo320 cells.

Results in this study suggest that msln expression is enhanced in CRC tissues and negatively correlated with the expression of hsa-mir-145-5p. However, this study is a retrospective clinical study with a small amount of samples. Hence, its clinical significance is limited to some extent. The large-scale experiments are still needed to further clarify and to provide a new theoretical basis for the diagnosis and treatment of colorectal cancer in the future.

## Figures and Tables

**Table 1:** Quantification of MSLN protein.

Peptide	Protein	cancer1	cancer2	normal1	normal2	para-carcinoma
DPSWRQPER	sp|Q13421|MSLN_HUMAN	9.96E+05	1.07E+06	8.19E+05	8.16E+05	9.97E+05
MSPEDIR	sp|Q13421|MSLN_HUMAN	1.87E+05	2.65E+05	5.24E+04	1.54E+05	4.13E+04
ALLEVNK	sp|Q13421|MSLN_HUMAN	4.65E+05	9.33E+05	2.02E+05	5.64E+05	1.48E+05
GALQNIIPASTGAAK	sp|P04406|G3P_HUMAN	1.53E+07	1.66E+07	1.20E+07	1.36E+07	1.18E+07
VIPELNGK	sp|P04406|G3P_HUMAN	2.59E+06	1.65E+06	2.58E+06	2.72E+06	1.30E+06
VPTANVSVVDLTCR	sp|P04406|G3P_HUMAN	2.80E+06	4.53E+06	4.22E+06	3.90E+06	3.45E+06
	MSLN	1.65E+06	2.27E+06	1.07E+06	1.53E+06	1.19E+06
	GAPDH	2.07E+07	2.28E+07	1.88E+07	2.02E+07	1.65E+07
	MSLN-normalization	1.66E+06	2.08E+06	1.18E+06	1.57E+06	1.49E+06

**Table 2:** Deregulated microRNAs in the inner tumor versus normal colorectal.

Upregulated microRNAs			Downregulated microRNAs		
microRNA	Q-Value	log2 fold change	microRNA	Q-Value	log2 fold change
hsa-miR-574-5p	0.0000584	3.60976505	hsa-miR-125b-5p	0.006924716	-2.1944318
hsa-miR-141-3p	0.000015	3.67688901	hsa-miR-145-5p	0.004944694	-3.4018351
hsa-miR-194-5p	0.00000154	3.04366356	hsa-miR-143-3p	0.000000784	-3.1709908
hsa-miR-200c-3p	0.0000132	3.62678268	hsa-miR-1-3p	0.000780008	-2.3017773
hsa-miR-4489	0.0000575	3.61068135	hsa-miR-1281	0.00000751	-2.0187418

## Data Availability

The raw data of gene expression for samples are available by emailing tegexibaiyin@yeah.net.

## Additional Points

*Strengths and Limitations of This Study*. (1) The literature shows that msln is one of the genes that are not expressed in normal tissues and are highly expressed after cancer occurs, and mir-145-5p has significantly low expression in various tumors. However, how msln and mir-145-5p are involved in tumors and whether or not there is a relationship between them are unclear, and reports related to colorectal cancer are even limited. (2) One significance of this study is that the expression and relationship of msln and mir-145-5p were analyzed in normal and cancerous colorectal tissues of patients with colon cancer. (3) In this study, *msln* was found to be a target gene of mir-145-5p in colorectal cancer. (4) We also found that mir-145-5p negatively regulates the proliferation of colorectal cancer colo320 cells and msln protein expression in colo320 cells. (5) We aimed to provide a new idea and basis for studying the diagnosis and treatment about colorectal cancer in the future. But, this study is a retrospective clinical study with a small amount of samples. Hence, its clinical significance is limited to some extent. Large-scale experiments are still needed for further research.
